# *“Because it eases my Childbirth Plan”*: a qualitative study on factors contributing to preferences for caesarean section in Thailand

**DOI:** 10.1186/s12884-023-05576-8

**Published:** 2023-04-24

**Authors:** Sasitara Nuampa, Ameporn Ratinthorn, Pisake Lumbiganon, Somporn Rungreangkulkij, Nilubon Rujiraprasert, Natthapat Buaboon, Nampet Jampathong, Alexandre Dumont, Claudia Hanson, Myriam de Loenzien, Meghan A. Bohren, Ana Pilar Betrán

**Affiliations:** 1 Department of Obstetrics and Gynaecological Nursing, Faculty of Nursing, Mahidol University, Bangkok, Thailand; 2 Department of Obstetrics and Gynaecology, Faculty of Medicine, Khon Kaen University, Khon Kaen, Thailand; 3 Centre for Research and Training on Gender and Women’s Health, Faculty of Nursing, Khon Kaen University, Khon Kaen, Thailand; 4 Department of Family of Nursing and Midwifery, Faculty of Nursing, Thammasat University, Prathumthani, Thailand; 5 Cochrane Thailand, Khon Kaen University, Khon Kaen, Thailand; 6 Université Paris Cité, IRD, Inserm, F-75006 Paris, Ceped France; 7 Department of Global Public Health, Karolinska Institutet, Stockholm, Sweden; 8 Gender and Women’s Health Unit, Centre for Health Equity, School of Population and Global Health, University of Melbourne, Carlton, VIC Australia; 9 UNDP/UNFPA/UNICEF/WHO/World Bank Special Program of Research, Development and Research Training in Human Reproduction (HRP), Department of Sexual and Reproductive Health and Research, World Health Organization, Geneva, Switzerland

**Keywords:** Caesarean section, Maternal health, Perception, Thailand, Women, Childbirth, QUALI-DEC

## Abstract

**Background:**

Although caesarean section (CS) rates have increased rapidly in Thailand, the upward trend is not supported by significant maternal or perinatal health benefits. The appropriate use of CS through QUALIty DECision-making by women and providers (QUALI-DEC project) aims to design and implement a strategy to optimize the use of CS through non-clinical interventions. This study aimed to explore the factors influencing women’s and health professionals’ preferences for CS delivery in Thailand.

**Methods:**

We conducted a formative qualitative study by using semi-structured in-depth interviews with pregnant and postpartum women, and healthcare staff. Purposive sampling was used to recruit participants from eight hospitals across four regions of Thailand. Content analysis was used to develop the main themes.

**Results:**

There were 78 participants, including 27 pregnant and 25 postpartum women, 8 administrators, 13 obstetricians, and 5 interns. We identified three main themes and seven sub-themes of women and healthcare providers’ perceptions on CS: (1) avoiding the negative experiences from vaginal birth (the pain of labor and childbirth, uncertainty during the labor period); (2) CS is a safer mode of birth (guarantees the baby’s safety, a protective shield for doctors); and (3) CS facilitates time management (baby’s destiny at an auspicious time, family’s management, manage my work/time).

**Conclusions:**

Women mentioned negative experiences and beliefs about vaginal delivery, labor pain, and uncertain delivery outcomes as important factors influencing CS preferences. On the other hand, CS is safer for babies and facilitates multiple tasks in women’s lives. From health professionals’ perspectives, CS is the easier and safer method for patients and them. Interventions to reduce unnecessary CS, including QUALI-DEC, should be designed and implemented, taking into consideration the perceptions of both women and healthcare providers.

**Supplementary Information:**

The online version contains supplementary material available at 10.1186/s12884-023-05576-8.

## Introduction

Caesarean section (CS) can be life-saving obstetrical surgical procedure in high-risk pregnancies [1]. However, the use of CS for delivery has continued its worrying rise worldwide. In particular, CS may be overused in middle-and high-income countries [2]. The significant reasons behind the high rate of CS are related to high socioeconomic status, availability and accessibility of the operation, types of private hospitals, and a shortage of health professionals in public hospitals [3–5]. Although the WHO does not recommend an “ideal” CS rate, CS rates exceeding 10% at a population level are not associated with reductions in maternal and newborn mortality, and exceeding 15% does not improve outcomes [2]. According to the most recent global estimates (2010–2018), 21.1% of women give birth via CS globally with averages ranging from 5% in Sub-Saharan Africa to 42.8% in Latin America and the Caribbean [6]. According to projections, unless effective interventions are implemented by 2030, 28.5% of women will give birth via CS globally, ranging from 7.1% in Sub-Saharan Africa to 63.4% in Eastern Asia [6]. In Thailand, a study of a hospital-based database of pregnant women and newborns under the Thai Universal Coverage Scheme of the National Health Security Office found that annual CS rates significantly increased from 23.2% to 2009 to 32.5% in 2017 [7]. The Thai Health Administrative Division reported that the CS rate nationwide increased to 43.2% in 2022 [8] and will continue to increase to 59.1% by 2030 [7]. These increasing trends are likely to be medically unnecessary, as they are not supported by significant maternal or perinatal health benefits [9].

CS is associated with an increased risk of adverse outcomes in women and babies [10, 11]. Pregnancy following cesarean delivery is associated with an increased risk of placenta previa, placenta accreta, and placental abruption [10]. CS is also associated with an increased risk of late childhood obesity and asthma [11]. In cases of medically necessary CS, benefits to the woman and/or baby may offset the risks. However, conducting a CS without medical indication exposes women and babies to unnecessary risks.

Both medical and non-medical factors affect women’s mode of birth. Non-medical factors include both the woman and her family’s beliefs and preferences, as well as the woman’s care environment and healthcare providers during pregnancy [12]. Globally, some women prefer birth by CS due to fears about labor pain, previous CS experience, and previous negative birth experience [13]. Regarding social factors, maternal preference for CS might also be driven by traditional customs of giving birth on a lucky or auspicious day [14]. Furthermore, factors associated with a preference for CS include a women’s older age, low education level, unemployment, smoking, symptoms of depression, and a history of abuse [13, 15, 16]. Increasing rates of CS in Thailand are likely influenced by health insurance plans covering the costs of CS. This may lead to the more comfortable decision of women and doctors for birth by CS, regardless of obstetric indications [17]. A study on the attitudes of pregnant women and obstetricians during antenatal care in Thailand showed that 87.5% of women and 68.9% of obstetricians preferred vaginal birth [18], while another study found that one-third of women need to participate in decision-making by choosing CS [19].

The views of healthcare providers, particularly obstetricians, significantly impact women’s mode of birth [20]. While some healthcare providers perceive the right of pregnant women to choose CS [21], some studies have found that CS is a conveniently scheduled procedure, less likely to result in litigation, and may generate more income than vaginal birth [22, 23]. As previously stated, the factors influencing CS preferences for both women and healthcare professionals have been discussed. In the context of sustained growing CS rates in Thailand, little is known about women’s and health providers’ perceptions of CS in the modern Thai context. Therefore, this study aimed to explore the factors influencing women and health professionals to prefer CS in Thailand. This formative study will provide better understanding of the contextual factors in Thailand that influence CS prior to implementing the QUALI-DEC project in Thailand.

## Methods

### Study design and setting

The QUALI-DEC project aims to design and implement a multifaceted strategy that is locally relevant, culturally accepted by women and providers, and can be implemented effectively to reduce unnecessary CS. In the first phase of the project, a formative study using a descriptive qualitative approach was conducted to elicit factors influencing preferences for CS in both women and health professionals [24, 25]. Semi-structured in-depth interviews (IDI) with pregnant women, postpartum women, and healthcare providers were conducted. This paper was reported according to the Consolidated Criteria for Reporting Qualitative Research (COREQ) Guidance [26].

The study was conducted in eight health facilities with CS rates ranging from 34.3 to 56.9% where the QUALI-DEC interventions will be implemented. The eight participating facilities were selected purposely according to the programmatic activities and priorities of the country and geographical representation of regions and facilities in Thailand with three hospitals in central Thailand, three in northeastern Thailand, one in northern Thailand, and one in eastern Thailand. All hospitals are public, with one secondary and seven tertiary hospitals.

### Participants, sampling, and recruitment

We identified three groups of participants: (1) pregnant women; (2) postpartum women; and (3) healthcare providers. The stratified sampling in this study recruited from eight hospitals consisted of 27 pregnant women, 25 postpartum women, 18 obstetric doctors, and 8 administrative doctors. The sample size was aligned with data saturation to ensure adequate data for drawing phenomenon conclusions [27].

The participants were purposefully selected from the antenatal care unit, postpartum unit, and obstetric medical department in each hospital. The inclusion criteria enrolled pregnant women aged 18 to 49 years with gestational ages between 28 and 42 weeks (nulliparous, multiparous women with a previous CS, and multiparous women without previous CS). Postpartum women were invited to participate in the study before discharge from the study hospitals, regardless of gestational age or mode of delivery. The exclusion criteria was women who could not read, write or understand Thai language. Healthcare providers who have worked for at least one year in the maternity unit were also invited to participate in this study. The eligible participants, which included 27 pregnant women, 25 postpartum women, 8 administrators, 13 obstetricians, and 5 interns, completed in-depth interviews.

### Data collection

The participants were recruited to participate in the interviews without coercion by a research assistant who was not hospital staff. Written informed consent was obtained from all participants before enrollment in the study. All interviews took place in private rooms in the hospitals without any distractions, and participants were alone with the interviewer during interviews. The interviews were set in several settings in the antenatal unit, postpartum unit, and staff office. Prior to the main interview questions, general discussion was started to build rapport. The 30-60-minute interviews were conducted in Thai and audio recorded. The participants were compensated with 500 baht (approximately USD $16) for their time. Data were collected between July and October 2020. All audio recordings were verbatim transcribed in Thai by a member of the research team. Transcripts that were anonymous were saved on a password-protected computer. Following the IDI, there was no additional contact with the research participants.

### Data collection instruments

An interview guide was developed based on the implementation of challenges identified in the WHO generic formative research protocol on optimizing the use of CS [28]. The guide was piloted and refined prior to data collection, then made available in the Additional file 1. In the interview guide, some of the topics focused on exploring CS preferences and related factors. In the case of the women, interview questions were used to explore values and needs regarding childbirth, prenatal information on mode of birth, and preferences. In the case of the professionals, their decision-making processes and perceived women’s preferences on mode of birth were revealed in addition to prenatal information provided.

### Data analysis

All qualitative data were analyzed with content analysis through a manual approach [25]. Five members of the research team [SN, AR, SR, NR and NB] who had various perspectives on the mode of delivery were involved in the data analysis. The researchers were separately immersed in the description and strict analysis of the transcriptions before encoding them. Data collection and analysis were conducted concurrently in order to ensure that new concepts emerging from the interviews could be explored in detail. In addition, field notes were taken immediately after the interviews to inform the researcher of important issues from the interviewer’s reactions. The verbatim transcription of the recorded interviews was read line-by-line and processed through open coding. Small clusters of codes were aggregated into broader ideas and more meaningful categories. The categories were sorted into themes by creating a tally sheet. Many of these items expressed similar ideas that could be formulated into themes [25, 29]. During the analysis process, the researchers met regularly to discuss the interpretive results by team consensus.

Regarding trustworthiness, confirmability was conducted through an audit trail using audio recordings that allowed the research team to check and recheck the data throughout the study. Field notes were taken immediately after the interviews to ensure contextual information. Rigorous content analysis and peer debriefing with the research team were conducted to confirm credibility. Member checking was used in the process of interviews to confirm the meaning of participants to ensure the credibility of the results [30]. The findings were reviewed during data analysis, and emerging findings were reported and discussed among the interviewer team. To preserve the original meaning, data analysis was undertaken in Thai, and excerpts from the interview transcripts in this article were translated by a bilingual Thai-English translator who is a part of the research team [31].

### Researcher characteristics and reflexivity

The QUALI-DEC research team consists of 14 Thai and 5 international researchers who are social scientists, nurses, doctors, and epidemiologists with expertise in maternal health. The IDIs were performed by five members of the Thai research team. All were female nursing professors with extensive experience in qualitative research for an average of five years and no prior relationship with any participants or work at the study site. Moreover, they were aware of the upward trend CS in Thailand is increasing, with numerous factors influencing decisions around the modes of birth. Furthermore, the team considered healthcare providers to be responsible for providing appropriate guidance on the mode of birth to support a safe pregnancy outcome. The research team was aware of these assumptions and kept them in mind throughout the study process to avoid any potentially adverse biases influencing participant responses or interpretations of results.

## Results

### Part 1: participant characteristics

A total of 78 participants participated (8 administrators, 13 obstetricians, 5 interns, 27 pregnant women, and 25 postpartum women). The characteristics of the pregnant and postpartum women participants are described in Table 1. The pregnant women ranged from between 18 and 41 years (median 27 years) in age. Gestational ages (GA) ranged from 29 to 39.1 weeks (median 35 weeks). The postpartum women ranged in age between 21 and 42 years (median 29 years). All postpartum women had full-term pregnancies. Seven women underwent intrapartum CS for the following medical reasons: cephalopelvic disproportion (n = 5) and fetal distress (n = 2). Five women underwent pre-labor CS due to maternal request (n = 2), maternal illness (n = 1), fetal macrosomia (n = 1), and breech presentation (n = 1).

The characteristics of the healthcare providers are described in Table 2. In addition, the eight administrators who were heads of departments ranged in age from 51 to 61 years (median 57.5 years). The median number of years working as a doctor was 30, ranging from 20 to 35 years. The obstetricians/interns had a median of 4.5 years of working experience as doctors or trainees within a range of 1 to 26 years.

**Table 1:** Characteristics of pregnant and postpartum women

	Pregnant women (N = 27)	Postpartum women (N = 25)
	n (%)	n (%)
**Age (year)**		
< 20 20–30 31–40 > 40	3 (11.1%) 14 (51.9%) 9 (33.3%) 1 (3.7%)	- 15 (55.6%) 9 (33.3%) 1 (3.7%)
**Marital status**		
Married Cohabitating Divorced	15 (55.6%) 11 (40.7%) 1 (3.7%)	17 (68.0%) 8 (32.0%) -
**Occupation**		
Employed Unemployed	19 (70.4%) 8 (29.6%)	17 (68.0%) 8 (32.0%)
**Parity**		
Primigravida ** Multigravida**	12 (44.4%) 15 (55.6%)	18 (72.0%) 7 (28.0%)
**Gestational age (week)**		
< 34 34.1–36.6 ≥ 37	10 (37.0%) 9 (33.3%) 8 (29.6%)	**-** **-** 25 (100%)
**Mode of birth**		
Vaginal birth Intrapartum CS Pre-labour CS: - Due to previous CS - Without previous CS	*N/A*	8 (32.0%) 7 (28.0%) 10 (40.0%) 5 (50.0%) 5 (50.0%)

**Table 2:** Characteristics of healthcare providers

	Administrators	Doctors/Interns
**Total number of participants**	8	18
**Gender**		
Female	2	12
Male	6	6
**Years working in total**		
1–5	0	7
6–10	0	5
11–15	0	2
16–20	1	2
21–25	0	1
26–30	4	1
≥ 31	3	0
**Years working at study facility**		
1–5	0	11
6–10	0	1
11–15	0	3
16–20	1	2
21–25	0	0
26–30	4	1
≥ 31	3	0

### Part 2: perceptions of pregnant women, postpartum women, and obstetricians about CS

The qualitative results in this study explored the perspectives of two stakeholders, women and health professionals, on factors influencing CS preferences. The pregnant and postpartum women disclosed their perspectives that CS is an easy childbirth plan because women’s lives during pregnancy and the transition to motherhood encounter several challenging tasks requiring balance and management. Regarding time management, CS facilitates time management and balances private lives among physicians as well. CS was also an option for women to avoid labor pain and uncertain childbirth outcomes, as well as being a likely safer mode of birth. According to healthcare providers, CS is an easy way to guarantee the baby’s safety. Vaginal birth, on the other hand, is regarded as more difficult to predict childbirth outcomes and necessitates high levels of competency and experience for clinical decisions and management. As a result, CS may protect them from being unsatisfied and prosecuted by women and their families. Table 3 shows three main themes and seven sub-themes identified by women and healthcare providers.

**Table 3:** Themes and sub-themes of the women and healthcare staffs’ perceptions on cesarean section

Theme	Sub-Theme	Frequency (women = 52, doctors = 26)
1. Avoiding the negative experiences from vaginal birth	1.1 Avoiding the pain of labor and childbirth	Pregnant women (18) Postpartum women (19)
	1.2 Avoiding uncertainty during the labor period	Pregnant women (9) Postpartum women (8) Admin (1) Obstetricians (2) Interns (3)
2. CS is a safer mode of birth	2.1 CS guarantees the baby’s safety	Pregnant women (7) Postpartum women (5) Admin (3) Obstetricians (5) Interns (1)
	2.2 CS is a protective shield for doctors	Admin (5) Obstetricians (4) Interns (4)
3. CS facilitates time management	3.1 Allowing me to decide on my baby’s destiny at an auspicious time	Pregnant women (6) Postpartum women (5)
	3.2 Facilitating my family’s management	Pregnant women (5) Postpartum women (5)
	3.3 Enabling me to manage my work/time	Pregnant women (1) Postpartum women (3) Admin (7) Obstetricians (7) Interns (4)

## Theme 1: avoiding the negative experiences from vaginal birth

The women in this study revealed their negative experiences and perceptions about vaginal birth in terms of fear of pain and uncertainty about vaginal birth outcomes. CS could help them avoid these experiences. There were two subthemes, as follows:

### Avoiding the pain of labor and childbirth

Fear of labor and childbirth pain was a major negative attitude toward vaginal delivery, which led to a preference for CS to avoid pain. Thirty-seven women believed CS as the only way to avoid the painful experiences of labor and vaginal birth, having had unpleasant previous experiences and perceptions with vaginal birth, particularly in encountering uncontrollable labor pain. These women did not feel equipped to cope with labor pain, and thus vaginal birth was viewed to be an unavoidable source of suffering and pain. This perspective of pain triggers women’s fear, and anxiety about vaginal birth. For example, a pregnant woman described her previous experience with vaginal birth, facing painful suffering. She emphasized that some healthcare providers told her that she had to tolerate labor pain.

*“I tried to stay calm, but it felt painful and uncomfortable when the uterus contracted every half an hour, 15 minutes, 5 minutes, and then every 1 minute. The time moved so slowly that it took a long time to be patient and forbidden to cry, which was excruciatingly painful for me.” (Pregnant woman 28 years, Multiparous without previous CS)*

A negative experience of pain from vaginal birth led the women to think about the advantages of a CS for future births:

*“It hurts!! The doctor wouldn’t allow me to push, it’s very painful…but the CS as I know it would not be painful during surgery. Comparing both methods [vaginal birth and CS], I think CS seems like a better way because it doesn’t make you suffer, right?” (Postpartum woman 29 years, Primiparous, Vaginal birth)*

Among these women, CS was viewed as a way to circumvent the pain of labor and vaginal birth. While they understood that post-CS healing could also be painful, CS could help them avoid pain during a longer labor.

### Avoiding uncertainty during labor

Women and doctors agreed that vaginal delivery was perceived as an uncertain and unpredictable method for childbirth in terms of labor duration, process, and outcome. While CS could provide a certain process of childbirth. Seventeen women agreed that CS helped them avoid uncertainty during delivery regarding the progress of labor, severity of pain, and “double suffering” from emergency CS. For example, one pregnant woman was afraid of getting double pain from both a failed vaginal birth and an emergency CS. The uncertain outcomes of vaginal birth may affect maternal perception of the mode of birth.

*“Unfortunately, my sister-in-law had some problems during childbirth and finally changed to a CS. I’m afraid I’ll have a similar problem if I choose to give birth vaginally. I will be in pain for 3–4 hours and again from the CS. Thus, I would definitely like to get a CS for sure. (Pregnant woman 38 years, Multiparous without previous CS)*

In addition, the women compared the unpredictable nature of labor duration to the shorter period of CS management.

*“…I’m still afraid of labor pain. For my last child 5 years ago, I felt pain from 6 pm until 10 pm. That was a long-suffering time…if you have a CS, you will know the exact time and the duration will be reduced.” (Postpartum woman 21 years, Multiparous without previous CS, Vaginal birth)*

Similarly, doctors disclosed that CS decisions in cases where there is an ambiguous indication for CS, such as cephalopelvic disproportion and a large infant, to avoid negative outcomes, vaginal delivery does not guarantee good fetal outcomes.

*“Without a doubt, I would prefer a CS. Attending during the labor period is difficult because we can’t predict the exact time and may encounter fetal distress.” (Medical doctor, work experience 18 years)*

## Theme 2: CS is a safer mode of birth

Both women and doctors agreed that CS was a safer mode of birth. In the case of pregnant and postpartum women, they expressed that CS is safer for their babies, while doctors agreed that it could guarantee the babies’ safety. Furthermore, the safe method for babies may shield doctors from patient dissatisfaction. Two subthemes were presented, as follows:

### CS guarantees the baby’s safety

Twelve women believed that the outcome of a CS is more certain about the child’s safety than vaginal birth. Infant safety was the most important aspect of CS preference revealed by beliefs and modern-era perspectives. CS was perceived as a quick method performed by highly-skilled physicians. Even though the women were aware of some risks of CS such as incision pain and the lengthy recovery period, they still felt that CS has fewer risks than vaginal delivery, which includes baby distress and trauma. For example, a pregnant woman said that her baby’s health was the most important consideration, emphasizing how delicate her baby was, as she said:

*“Right now, the health of my child is the most important aspect of my pregnancy. I didn’t give any consideration to myself because I believe I can handle it. But not my child, and I just want him to be safe.” (Pregnant woman 29 years, Multiparous with previous CS)*

Furthermore, the doctor’s competence in performing CS was an important factor related to women’s trust.

*“CS is sure for the baby’s health. I saw some women on social media who were depressed because of fetal distress caused by vaginal birth*… *for CS, I am sure the doctor’s skills can help my baby safely.” (Pregnant woman 29 years, Multiparous with previous CS)*

Many doctors were also concerned about the risks of vaginal birth during the intrapartum period, particularly the risk of infant complications, which could lead to blame, lawsuits, and stigma. The CS would be a safer method of childbirth than vaginal birth, requiring fewer skills and greater precision. Three administrators, five doctors, and one intern believed that vaginal birth was difficult to manage due to the unpredictable length of time it takes for the baby to be born. In addition, there may be complications in the birthing process, such as labor obstruction, severe perineal laceration, hemorrhage, the baby’s head trauma, or any danger from birth. A doctor talks about his friend facing a negative consequence of vaginal birth, and reflected the opposite side of the risk of vaginal birth.

*“…my friend attended to a shoulder dystocia case and, unfortunately, the baby died. He quit his job to work only on medical record, not clinical practices anymore.” (Medical doctor, work experience 20 years)*

A vaginal birth safely includes the need for precise skills in labor progress assessment, timely assessment of complications, and effective management.

*“There will be problems such as some cases...there is a chance of causing hematoma or tearing down to the anal sphincter as well. There might be some things beyond our control. If we make a mistake in the assessment, that is the disadvantage.” (Resident, work experience 6 years)*

Therefore, some healthcare providers perceived CS as a safer mode of birth compared to vaginal birth. In some cases CS may reduce the incidence of injury to the baby. Finally, women could avoid labor pain, resulting in more satisfaction than vaginal birth.

### CS is a protective shield for doctors

The CS protected the physician, implying that CS delivery may be appropriate care for meeting the needs of patients and families, as well as a reasonable procedure in high-risk situations. Similarly, CS can protect the clinician against dissatisfied patients. Along with advancing medical technology, CS is viewed by some doctors as a straightforward and convenient mode of birth. When there are complications with vaginal birth, healthcare providers, particularly obstetricians, were reportedly quick to perform a CS, as they believed it to be safer and easier than instrumental birth using vacuum or forceps. Furthermore, thirteen healthcare staff members believed that CS met the requirements of women and their families while preventing litigation in the event of negative birth outcomes. One doctor mentioned;

*“If fetal distress is detected only partially, we may decide to perform a CS right away out of concern for negative outcomes in later shifts, which could lead to legal issues.” (Medical doctor, work experience 11 years)*

Moreover, it is easy to perform CS, while using other modes of instrumental birth such as vacuums or forceps may require expertise and high-level skills. Thus, CS is the best solution to prevent adverse outcomes.

*“We work on women and their families’ expectations, right? If there is a little obstruction to vaginal birth, we rapidly do something for a solution, such as intrapartum CS. For assisted procedures such as vacuum extraction, it is more difficult” (Medical doctor, work experience 10 years)*

## Theme 3: CS facilitates time management

CS was perceived as a mode of childbirth that facilitated time management in various key aspects of life for both women and doctors. Pregnant and postpartum women expressed their perceptions and experiences in terms of how CS facilitated planning an exact date and time to balance their maternal role and work, as well as cultural beliefs. For healthcare providers, CS could provide the precise schedules they need and allow them to manage their many duties in the workplace. There were three subthemes, as follows:

### Allowing me to decide on my baby’s destiny at an auspicious time

The auspicious time was a critical point of view linked with cultural beliefs predicting the destiny of an infant. The birth date would represent the best start to the baby’s life and meet the family’s expectations, which could be facilitated by CS delivery. Eleven women who preferred CS believed that it could allow them to schedule the birth for an auspicious time. They could choose the best birth times to predict that the baby’s life will be good in the future. For example,

*“In my mind, I prefer CS since I can schedule the good and ready days in advance. It will help me feel less anxious. I have the ability to choose my own day and time.” (Pregnant woman 38 years, Multiparous without previous CS)*

Furthermore, the influence of social media and the significant other’s experiences may influence women’s preference for CS.

*“Now, there are many media on the internet. So, many people view that CS is normal. From my friend’s perspective, most of them choose CS because they could control their baby’s destiny, deciding on an auspicious time for CS.” (Pregnant woman 34 years, Nulliparous)*

### Facilitating my family’s management

CS aids family management, demonstrating interpersonal influence on the mother’s mode of delivery decision. Women with CS can plan and manage their schedules with their families, relieving stress and organizing comprehensive postpartum preparation. From a logistical perspective, CS is perceived as a convenient and controllable birth mode for women and their families. In this study, ten women and their families had positive perspectives about CS, believing that CS best met women’s and families’ needs as a solution to reconcile life and maternal demands. For example, a woman spoke about the need to consider practical aspects such as the necessity of time management in preparing materials, caregiver, and maternal responsibilities for child care. Thus, CS makes her plan easier.

*“I may not know how to go to the hospital, who will care for me while I’m there, or what vital items I’ll need because I don’t know the exact time for birthing. I also have to handle my son’s daily routine when he goes to school... I can’t tell when labor pain will start.” (Postpartum woman 34 years, Multiparous, intrapartum CS)*

### Enabling me to manage my work/time

CS has significant professional and social meaning. In their daily lives, women have to manage multiple tasks. The organizational factor influences CS delivery preference, which facilitates work responsibility and the maternal role. Three women described how CS could help them manage their full-time working in urban areas. For example, a woman may be concerned about her work responsibilities as she attempts to manage and balance both her maternal and societal roles.

*“I had already planned an auspicious time for my maternal leave and managed my work.” (Postpartum woman 30 years, Primiparous, Pre-labor CS)*

The CS was able to assist women in balancing their lives and social and professional relationships.

“*Actually, my boss allowed me to stop working and take maternity leave. But I worry about my work…CS can help me have certainty in my schedule.” (Pregnant woman 25 years, Nulliparous)*

Regarding healthcare providers’ viewpoints, CS can help them manage their clinical and academic work and personal time. Clinical time management allowed to figure out an organization’s influence and work system in hospitals. Busy schedules and many duties in the workplace could affect the quality of life of health professionals. Seven administrators, seven obstetricians, and four interns indicated that CS allowed them more precision to determine the time for birth, manage time for other clinical work, and balance their personal lives. The time management concern is connected to staff shortages, large numbers of patients, multiple jobs, and staff health. For example, a doctor believed that the advantage of CS was that it is better and faster:

*“CS is better and faster…vaginal birth is more likely to increase the risk if the mother has pushed for a long time. The child who has been stuck for a long time will have more hypoxia because of lack of oxygen and caput succedaneum than CS.” (Resident, work experience 4 years)*

Furthermore, the effectiveness of time management was influenced by the organizational system. The barriers were frequently discovered to be a result of a staff shortage. As a result, CS could be handled quickly.

*“You must comprehend the setting of our facility. We receive 500–600 cases per month, despite having only six obstetricians. We put up so much effort! It’s easy to make decisions on unclear indication of CS such as cephalopelvic disproportion. After a job is done, we can take a rest.” (Medical doctor, work experiences 20 years)*

## Discussion

This qualitative study deepens our understanding of women’s and healthcare providers perceptions of the benefits of CS compared to vaginal birth in Thailand. These benefits vary between women and healthcare providers, but are typically centered on the convenience of CS to schedule birth, avoid labor pain, and ensure safe childbirth. In addition, these results allow us to better understand some of the most important values in Thai society likely driving increasing rates of CS in this country, particularly in relation to CS on maternal request.

Most women in this study shared their concerns about fear of labor pain and uncertainty during vaginal birth, which resulted in preferring CS as a way to avoid both. Women’s negative experiences with these reasons have been represented in stories aired in the mass media and within communities. Storytelling about childbirth experiences from mother-to-mother providing not only information, but also a means for empathy and understanding [32]. Several studies found that fear of pain could predict a preference for elective CS [22, 33–35]. On the other hand, women who preferred vaginal birth in relation to the positive experience of childbirth and the belief that vaginal birth is an essential part of being a woman and mother as the best initiation into motherhood [36, 37]. There were potential strategies for promoting positive experience from vaginal birth, including antenatal information about mode of delivery, labor companionship, and labor anesthesia [38–43]. In government hospitals, the antenatal clinic is often very busy, leaving inadequate time for women to discuss their fears and concerns about labor and vaginal birth with healthcare providers. Antenatal classes for childbirth preparation are also lacking in Thailand. Research conducted in other countries has found that antenatal education could increase childbirth self-efficacy, coping with labor pain, greater perceived support and control in birth with less fear of birth and post-traumatic stress disorder symptoms following childbirth [38–40]. Moreover, labor companionship has important benefits for positive childbirth experiences. Companions can be a bridge over communication gaps between health workers and women in addition to facilitating non-pharmacological pain relief [41]. Regarding pain management with labor analgesia, epidural analgesia is associated with greater pain relief than non-epidural methods [43]. However, the women in this study did not mention epidural analgesia during labor for reducing pain, which might be a limitation of medical resources and accessibility. Moreover, this study included a number of women who had previously experienced perinatal death, which might have had an impact on their childbirth experience.

In previous studies, there were a few discussions about CS and women’s social roles and responsibilities. This study found that the reality of women’s social and professional roles was challenging and complex, particularly for working women. Women’s scheduling a CS allows them to better organize and plan other responsibilities. In Thai culture, most women have to balance many tasks and live in an unequal power relationship between genders. As the head of the family, men are not responsible for the household duties, and women have to manage the household, childcare, and outside work [44]. Moreover, Thai women have to balance a range of roles because of prescribed gender norms that make it challenging to develop new employment opportunities [45]. Similarly, the study of Arghavanian et al. [46] found that women who were pregnant and employed faced challenges navigating the gender rules of the workplace and home environments, and many struggled to maintain roles as employees, mothers and/or wives. Moreover, some women who preferred CS had positive views on possibility to plan day and time scheduling [47, 48]. Regarding the complexity of the situation, the results suggest that health providers should apply counseling skills to listen more to their difficult lives, beliefs, sources of support, and social roles that may influence women’s decisions on birth methods. Moreover, family participation at the beginning of the program may provide support and allow women to balance their roles.

Moreover, several women and healthcare providers in this study perceived positive viewpoints on CS as the childbirth method to give safe birth to babies. According to previous studies, CS is safer for baby and/or mother, which is a key factor influencing the preference for CS [35, 49]. Women do not seem to be aware of the risks of CS to their babies’ or their own health, either in the short or long term. Studies have shown that particularly long-term risks are less acknowledged and spread [50–52]. Women with previous CS have described a lack of information on birthing options affecting their childbirth decision-making [53]. Another study found the majority of women to have very little information about their mode of delivery from healthcare professionals [54]. Antenatal education is also important to appropriately inform women about the potential benefits and harms associated with different delivery modes. Moreover, this is a suitable period for conducting a prenatal childbirth class emphasizing the methods for reducing labor pain and increasing the chances of positive childbirth experiences.

From obstetricians’ viewpoints, CS is perceived as a positive and necessary management for high-risk cases to ensure the safety of infants during an emergency and prevent patient dissatisfaction. However, the medical indications and clinical practice guidelines for necessary CS should be declared and discussed. According to a study in China, health professionals believed CS to be safe due to its availability and accessibility [45, 55]. In contrast to the quality of vaginal delivery, healthcare professionals were critical of the lack of skills and training available for vaginal birth as a consequence of the increasing use of CS [45]. Likewise, this study found that negative perceptions of vaginal birth were related to experiences of adverse outcomes and limited experience and skills with assisted vaginal birth to manage complications during labor. The study of Parás et al. [56] revealed that 60.9% of obstetricians perceived being skillful at CS; 35% had scheduled a CS for convenience, and 83.8% believed that women prefer CS. The issue of physician skills for management in vaginal birth and using instrumental vaginal birth may require doctors to train or re-train in obstetric skills in order to increase doctors’ confidence and confirm positive vaginal birth outcomes for now and in the future [57, 58]. The support system for staff competencies at the hospital and national levels should be set up effectively.

## Implications for practice and research

Our results will be used to develop and tailor effective interventions to reduce unnecessary CS by addressing the negative perceptions of vaginal birth among both women and doctors in Thailand. Moreover, we identified limitations in knowledge about benefits and risks of different modes of birth among women. These results support and guide antenatal intervention with a data analysis tool (DAT) providing comprehensive childbirth information and a draft on how to use DAT with counseling skills and preparing pain management for optimal outcomes. For obstetricians, the training system on necessary childbirth skills may increase practical confidence and create a support system to help them balance their work and lifestyles.

## Conclusion

From both women’s and doctors’ viewpoints, CS is seen as a safer mode of birth for the baby and facilitate time management compared to vaginal birth. In the case of women, CS was primarily perceived as having the potential to help them avoid labor pain and uncertainty during labor. Moreover, CS allows women to manage their time for family management and balance their work, as well as adhere to cultural beliefs. Furthermore, obstetricians’ perspectives of CS were that it was simple to manage clinical time. The intervention to reduce unnecessary CS should be designed and implemented by concerning both women’s and doctors’ perceptions.

### Electronic supplementary material

Below is the link to the electronic supplementary material.

Additional file 1

## Data Availability

The datasets used and/or analysed during the current study are available from the corresponding author on reasonable request.
