# Correction: “Because it eases my childbirth plan”: a qualitative study on factors contributing to preferences for caesarean section in Thailand

**DOI:** 10.1186/s12884-024-06379-1

**Published:** 2024-03-14

**Authors:** Sasitara Nuampa, Ameporn Ratinthorn, Pisake Lumbiganon, Somporn Rungreangkulkij, Nilubon Rujiraprasert, Natthapat Buaboon, Nampet Jampathong, Alexandre Dumont, Claudia Hanson, Myriam de Loenzien, Meghan A. Bohren, Ana Pilar Betrán

**Affiliations:** 1 Department of Obstetrics and Gynaecological Nursing, Faculty of Nursing, Mahidol University, Bangkok, Thailand; 2 Department of Obstetrics and Gynaecology, Faculty of Medicine, Khon Kaen University, Khon Kaen, Thailand; 3 Centre for Research and Training on Gender and Women’s Health, Faculty of Nursing, Khon Kaen University, Khon Kaen, Thailand; 4 Department of Family of Nursing and Midwifery, Faculty of Nursing, Thammasat University, Prathumthani, Thailand; 5 Cochrane Thailand, Khon Kaen University, Khon Kaen, Thailand; 6 Université Paris Cité, IRD, Paris, Ceped, F-75006 Inserm France; 7 Department of Global Public Health, Karolinska Institutet, Stockholm, Sweden; 8 UNDP/UNFPA/UNICEF/WHO/World Bank Special Program of Research, Development and Research Training in Human Reproduction (HRP), Department of Sexual and Reproductive Health and Research, World Health Organization, Carlton, VIC Australia; 9 Gender and Women’s Health Unit, Centre for Health Equity, School of Population and Global Health, University of Melbourne, Geneva, Switzerland

**Correction**: ***BMC Pregnancy Childbirth*23, 280 (2023)**

10.1186/s12884-023-05576-8.

Following publication of the original article [1], the authors reported an error in Ethics approval and consent to participate section.

The current information states “Udon Thani Hospital (UDH REC No 28/2564)” which should be amended to “Udon Thani Hospital (UDH REC No 28/2563). ”

The original article has been corrected.
