# Supplementary material for: “Because it eases my Childbirth Plan”: a qualitative study on factors contributing to preferences for caesarean section in Thailand
Source: BMC Pregnancy Childbirth. 2023 Apr 24;23:280. doi: 10.1186/s12884-023-05576-8 (PMC10124050; doi:10.1186/s12884-023-05576-8)
Supplement: Supplementary file 1 — Additional file 1 [file 12884_2023_5576_MOESM1_ESM.pdf]

In-depth interview: pregnant and postpartum women

Step 1: Introduce yourself to the participant. Describe the purpose of the interview and how information will be used. Obtain oral/written consent.
Step 2: Ask the participant to identify herself. Interviewer: fill out the information below prior to beginning the interview.
Step 3: Conduct the interview. Please remember to audio record the interview.

Interview place: ________________________________

Interview date: _________________________________

Start time: ____________________________________

End time: _____________________________________

Interviewer name: _________________________________

Interview identification: ____________________________________

Participant information

Duration of current pregnancy (weeks/months, please label):________________________________

Age (years): ____

Marital status (Single, married/cohabitating, divorced, widowed): _____________________

Occupation (write in): ____

Part 1. Values and needs surrounding the childbirth period

1. What are some things you are doing to prepare for your birth?
    a. Probe: who is helping you to prepare? How are they helping?

2. Thinking about your pregnancy, what are some of the things you value most? Why?
    a. Probe: to keep yourself healthy?
    b. Probe: to keep your baby healthy?

3. Thinking to the future about your birth, what are some of the things that are most important to you?
    a. Probe: What will you need from your doctors and midwives to make sure this happens?
    b. Probe: what will you need from your family to make sure this happens?
    c. Where are you planning to give birth? Why?
        i. Did anyone help you decide where to give birth? Who? How did they help?

Part 2. Prenatal education

4. Thinking about when you go to your antenatal care visits, what are some of the things that are most important to you to learn about?
    a. Probe: What are some of the most important things you have learned during antenatal care? Why are they important?
    b. Probe: How do you think antenatal care could be improved?
        i. What do you think is missing from your antenatal care visits?
        ii. Are there any things that you would remove or change during your antenatal care visits?

Part 3. Preferences and decision-making processes regarding mode of childbirth

5. Could you tell me about the different ways that women can give birth?
    a. Probe: How did you learn about these options?

6. What do you think about vaginal birth and caesarean section?

7. What do you think are some of the positive things about vaginal birth?
    a. Probe: Why are these positive things?
    b. Probe: How did you learn about these positive things?

8. What are some of the negative things about vaginal birth?
    a. Probe: Why are these negative things?
    b. Probe: How did you learn about these negative things?

9. What do you think are some of the positive things about caesarean section?
    a. Probe: Why are these positive things?
    b. Probe: How did you learn about these positive things?

10. What are some of the negative things about caesarean section?
    a. Probe: Why are these negative things?
    b. Probe: How did you learn about these negative things?

11. <u>How did you learn about vaginal birth and caesarean section?</u>

12. <u>How would you prefer to give birth? For example, caesarean or vaginal birth?</u>
    a. Why do you prefer to give birth this way?
    b. How important is it for you to give birth this way? Why?
    c. Was anyone involved in helping you make a decision about how you prefer to give birth?
        i. Probe: Will your (husband/partner) influence this decision? How?
        ii. Probe: Will your family influence this decision? How?
        iii. Probe: Will your friends influence this decision? How?
        iv. Probe: Will your doctor or midwife influence this decision? How?
        v. Probe: Will the media influence this decision? How?

13. <u>Do you feel like you have enough information to understand the options that you have for how to give birth? Why or why not?</u>
    a. <u>Probe: What other type of information about different modes of childbirth would you be interested to learn about?</u>
    b. <u>At what point during your pregnancy would you like to receive this information?</u>

14. Did you discuss your preference with your doctor or midwife? If so, what was the discussion like?

15. *A decision-analysis tool could help to educate women about their options for mode of birth and how to discuss their preferences with a doctor. Interviewer: show the woman the Vietnam decision analysis tool.*
    a. Would this type of tool be helpful to you? Why or why not?
    b. What type of information would you like to have included?
    c. *This type of decision tool can be paper based like this example, or could be an application for a phone*. Which of these options do you prefer and why?

16. <u>Pregnancy and childbirth are exciting times but can also be scary for some women. Is there anything that you are afraid or nervous of during your pregnancy? Why or why not?</u>
    Note to interviewer: if they bring up fear of pain, then probe about what pain management technique they have learned about.

    a. What about during your birth, is there anything that you are afraid of? Why or why not?
        i. Have you spoken to your doctor or midwife about these fears? Why or why not? What did they tell you?
        ii. Have you spoken to anyone else about these fears?
            1. <u>*If yes*</u>:
                a. Who did you speak to? Why did you choose to speak to this person?
                b. What type of advice did they give you?
            2. <u>*If no*</u>: Do you plan to speak to anyone about these fears? Why or why not?
        iii. What do you think could be done to help reduce this fear for you?

Part 4. Labour companionship

17. <u>What do you need in order to have a positive experience when you go to the hospital for childbirth?</u>
    d. What type of support do you think you need during labour and childbirth?

Interviewer read: A labour companion is a person of the woman's choice, who can help to provide emotional support to the woman during labour and childbirth. Typically, this person would be with the woman continuously throughout labour and childbirth. This person may be the woman's husband/partner, her mother, or a friend.

18. Do you think you will receive this type of support? Why or why not?

19. <u>Have you ever heard of someone providing this type of support?</u>

20. <u>What do you think of this type of support?</u>

21. <u>Do you know if labour companionship is allowed in the hospital you plan to give birth in?</u>
    e. *If labour companionship is not allowed*: What do you think are the reasons for not allowing a labour companion?
    f. Would you be allowed a labour companion if you requested it? Why or why not?
    g. In your opinion, what changes do you think the hospital could make in order to make it more comfortable for women to have a labour companion?

22. <u>Do you think you would want to have a labour companion for your upcoming birth? Why or why not?</u>
    h. What type of information or education would YOU need before deciding if you wanted to have a labour companion to support you?

23. <u>If you were to have a labour companion with you:</u>
    i. What would you expect from this person?
    j. When would you want this person to be with you in the hospital (probe: the whole time, only during labour but not during the birth, something else?)
    k. Who would you prefer this person to be? Why?
    l. When would you like to start talking to your labour companion about their role during your labour and childbirth?
  iii. Probe: at what month during your pregnancy?

24. <u>What type of information or education do you think a labour companion would need to be able to support you?</u>

Thank you so much for your time. Is there anything else that you would like to share with me today about anything we talked about?
